# Natural Human Infections with *Plasmodium cynomolgi*, *P. inui*, and 4 other Simian Malaria Parasites, Malaysia

**DOI:** 10.3201/eid2708.204502

**Published:** 2021-08

**Authors:** Nan Jiun Yap, Hanisah Hossain, Thamayanthi Nada-Raja, Romano Ngui, Azdayanti Muslim, Boon-Peng Hoh, Loke Tim Khaw, Khamisah Abdul Kadir, Paul Cliff Simon Divis, Indra Vythilingam, Balbir Singh, Yvonne Ai-Lian Lim

**Affiliations:** Universiti Malaya, Kuala Lumpur, Malaysia (N.J. Yap, R. Ngui, A. Muslim, I. Vythilingam, Y.A.-L. Lim);; Universiti Malaysia Sarawak, Kota Samarahan, Sarawak, Malaysia (H. Hossain, T. Nada-Raja, K.A. Kadir, P.C.S. Divis, B. Singh);; Universiti Teknologi MARA (Sungai Buloh Campus), Selangor, Malaysia (A. Muslim);; UCSI University, Kuala Lumpur, Malaysia (B.-P. Hoh);; International Medical University, Kuala Lumpur, Malaysia (L.T. Khaw)

**Keywords:** *Plasmodium*, simian, malaria, zoonoses, parasites, Malaysia, *Plasmodium cynomolgi*, *Plasmodium inui*

## Abstract

We detected the simian malaria parasites *Plasmodium knowlesi*, *P. cynomolgi*, *P. inui*, *P. coatneyi*, *P. inui*–like, and *P. simiovale* among forest fringe–living indigenous communities from various locations in Malaysia. Our findings underscore the importance of using molecular tools to identify newly emergent malaria parasites in humans.

Zoonotic malaria caused by *Plasmodium knowlesi*, commonly found in long-tailed macaques (*Macaca fascicularis*) and pig-tailed macaques (*M. nemestrina*), is now a major emerging disease, particularly in Malaysia (*1*,*2*). Two other simian malaria parasites, *P. cynomolgi* (*2**–**4*) and *P. inui* (*2*), have also been shown to have the potential of zoonotic transmission to humans through the bites of infected mosquitoes under natural and experimental conditions. The risk of acquiring zoonotic malaria is highest for persons living at the forest fringe and working or venturing into the forest because of their proximity with the monkey reservoir hosts and the mosquito vectors (*5*,*6*). With the aid of molecular methods, we aimed to investigate whether human infections with simian malaria parasites were present among indigenous communities in Malaysia whose villages are situated in the forest or at the forest fringe.

## The Study

We examined 645 archived blood samples that we had collected during 2011–2014 among indigenous populations of various subtribes from 14 villages in 7 states in Malaysia (Appendix Table 1). We first screened the extracted DNA samples at Universiti Malaya (UM) for the presence of *Plasmodium* with the aid of genus-specific primers (rPLU1 and rPLU5; rPLU3 and rPLU4) (Appendix). Of the 645 indigenous community samples, 102 (15.8%) were positive for *Plasmodium*. Using species-specific nested PCR assays (Appendix), we identified these infections as monoinfections with *P. knowlesi* (n = 40), *P. vivax* (n = 21), *P. cynomolgi* (n = 9), *P. falciparum* (n = 6), *P. coatneyi* (n = 3), *P. inui* (n = 3), *P. malariae* (n = 2), and *P. ovale curtisi* (n = 1) (Table 1). In 17 samples, the species could not be identified despite repeated attempts. Our species-specific primer pairs were designed on the basis of either the asexually (A) or sexually (S) transcribed forms of *Plasmodium* small subunit (SSU) rRNA genes (*7*); the genus-specific primer pairs anneal to both asexual and sexual forms of the SSU rRNA genes, and therefore the genus-specific assay is more sensitive.

**Table 1:** Human and simian *Plasmodium* malaria species identified by nested PCR at UM targeting SSU rRNA genes among indigenous community blood samples, by state, Malaysia*

State	No. samples tested	No. positive samples	Human and simian malaria species							
			*P. falciparum*	*P. vivax*	*P. malariae*	*P. ovale curtisi*	*P. knowlesi*	*P. coatneyi*	*P. cynomolgi*	*P. inui*
Pahang	109	5	0	2	0	1	2	0	0	0
Perak	61	55	3	10	2	0	26	3	5	0
Selangor	49	0	0	0	0	0	0	0	0	0
Negeri Sembilan	163	13	1	2	0	0	2	0	2	0
Melaka	32	13	2	3	0	0	1	0	1	1
Kelantan	32	9	0	2	0	0	6	0	1	0
Sarawak	199	7	0	2	0	0	3	0	0	2
Total/overall prevalence	645	102† (of 645; 15.8%)	6 (of 102; 5.9%)	21 (20.6%)	2 (2.0%)	1 (1.0%)	40 (39.2%)	3 (2.9%)	9 (8.8%)	3 (2.9%)

*SSU, small subunit; UM, Universiti Malaya. †102 of 645 (15.8%) indigenous community samples were found positive with *Plasmodium* genus-specific primers; 17 *Plasmodium* genus-positive samples could not be identified up to species level despite repeated attempts.

**Figure 1:**
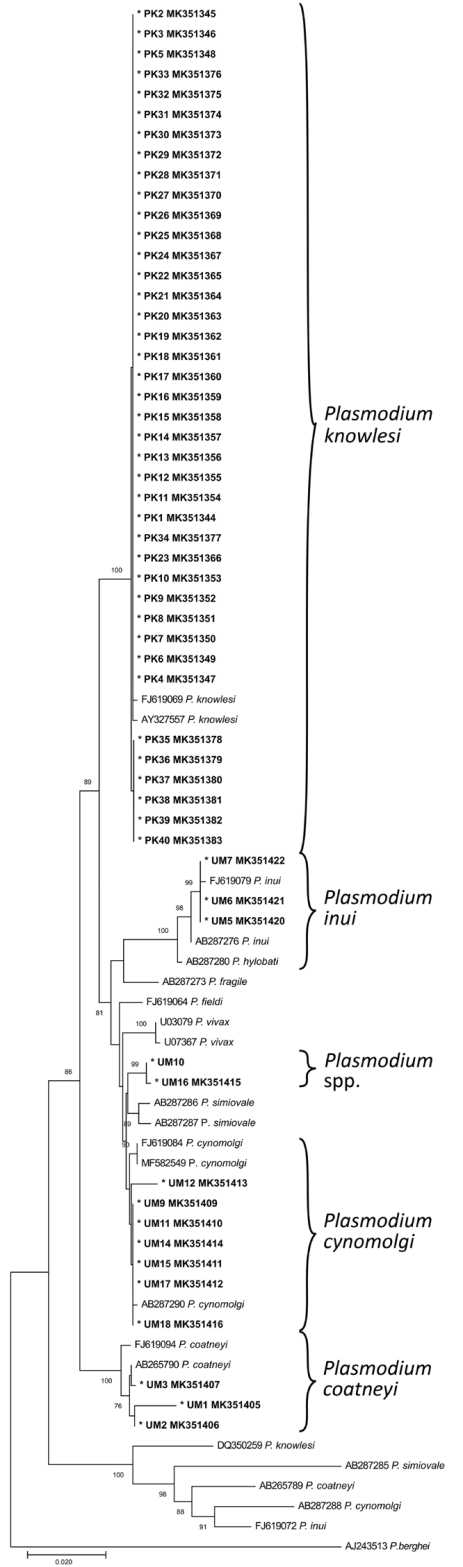
Neighbor-joining phylogenetic tree of *Plasmodium* species based on partial sequence of SSU rRNA genes for identification of *Plasmodium* malaria species from indigenous community blood samples, Malaysia. Nucleotide sequences generated from this study are marked with asterisks and are in bold. GenBank accession numbers are provided for all sequences. Numbers at nodes indicate percentage support of 1,000 bootstrap replicates; only bootstrap values above 70% are displayed. Scale bar indicates branch length.

We further characterized the 55 samples that tested positive for simian malaria parasites by amplifying a longer fragment of the SSU rRNA gene (914 bp–950 bp) for direct sequencing. Phylogenetic analysis using the neighbor-joining method (Figure 1) revealed the presence of *P. knowlesi* (samples PK1–40), *P. coatneyi* (UM1–3), *P. cynomolgi* (UM9, UM11, UM12, UM14, UM15, UM17, UM18), and *P. inui* (UM5–7). Meanwhile, 2 sequences derived from samples UM10 and UM16 were found to be closely related to *P. simiovale*.

We then reextracted DNA from 15 blood samples that were positive for *P. coatneyi*, *P. cynomolgi*, and *P. inui* and sent these samples (blinded) together with 5 *Plasmodium*-negative samples to Universiti Malaysia Sarawak (UNIMAS) to confirm their identities by PCR and sequencing of part of the cytochrome c oxidase subunit 1 (COX1) gene. At UNIMAS, using nested PCR assays based on SSU rRNA genes, we found 1 single and 9 double species infections. We could not identify the species of *Plasmodium* for sample UM6, 4 of the *Plasmodium*-positive samples from UM were *Plasmodium* negative, and all 5 *Plasmodium*-negative samples from UM (UM4, 8, 13, 19, 20) tested negative (Table 2). Furthermore, because both laboratories at UM and UNIMAS had previously extracted DNA from macaque blood to examine for simian malaria parasites, we tested the samples for macaque DNA to rule out the possibility that the simian malaria parasites detected were the result of contamination with macaque blood. We obtained negative results using nested PCR for detection of macaque DNA for the 20 DNA samples when they were first received at UNIMAS and also when we repeated testing after completing the sequencing of COX1 genes, indicating that these samples were not contaminated with macaque blood upon receipt or during subsequent experiments at UNIMAS.

**Table 2:** Comparison between results of nested PCR and sequencing at UM and UNIMAS for identification of *Plasmodium* malaria species from indigenous community blood samples, Malaysia*

Sample ID	Identification at UM			Identification at UNIMAS	
	PCR assays based on SSU rRNA genes	Phylogenetic analysis of SSU rRNA genes		PCR assays based on SSU rRNA genes	Phylogenetic analysis of COX1 genes
UM1	*P. coatneyi*	*P. coatneyi*		Negative	ND
UM2	*P. coatneyi*	*P. coatneyi*		Negative	ND
UM3	*P. coatneyi*	*P. coatneyi*		Negative	ND
UM5	*P. inui*	*P. inui*		Negative	ND
UM6	*P. inui*	*P. inui*		Positive	*P. inui*–like, *P. simiovale*
UM7	*P. inui*	*P. inui*		*P. inui*	*P. inui*-like
UM9	*P. cynomolgi*	*P. cynomolgi*		*P. cynomolgi, P. inui*	*P. cynomolgi*
UM10	*P. cynomolgi*	*Plasmodium* spp.		*P. cynomolgi, P. inui*	*P. cynomolgi*
UM11	*P. cynomolgi*	*P. cynomolgi*		*P. cynomolgi, P. inui*	*P. cynomolgi*
UM12	*P. cynomolgi*	*P. cynomolgi*		*P. cynomolgi, P. inui*	*P. cynomolgi*
UM14	*P. cynomolgi*	*P. cynomolgi*		*P. cynomolgi, P. inui*	*P. cynomolgi*
UM15	*P. cynomolgi*	*P. cynomolgi*		*P. cynomolgi, P. inui*	*P. cynomolgi*
UM16	*P. cynomolgi*	*Plasmodium* spp.		*P. cynomolgi, P. inui*	*P.* *cynomolgi*, *P. inui*–like, *P. simiovale*
UM17	*P. cynomolgi*	*P. cynomolgi*		*P. cynomolgi, P. inui*	*P. cynomolgi*
UM18	*P. cynomolgi*	*P. cynomolgi*		*P. cynomolgi, P. inui*	*P. cynomolgi*

*Negative, negative for *Plasmodium* DNA and not examined by species-specific nested PCR assays; ND, not done; positive, positive for *Plasmodium* DNA but negative with species-specific nested PCR assays. SSU, small subunit; UM, Universiti Malaya; UNIMAS, Universiti Malaysia Sarawak.

**Figure 2:**
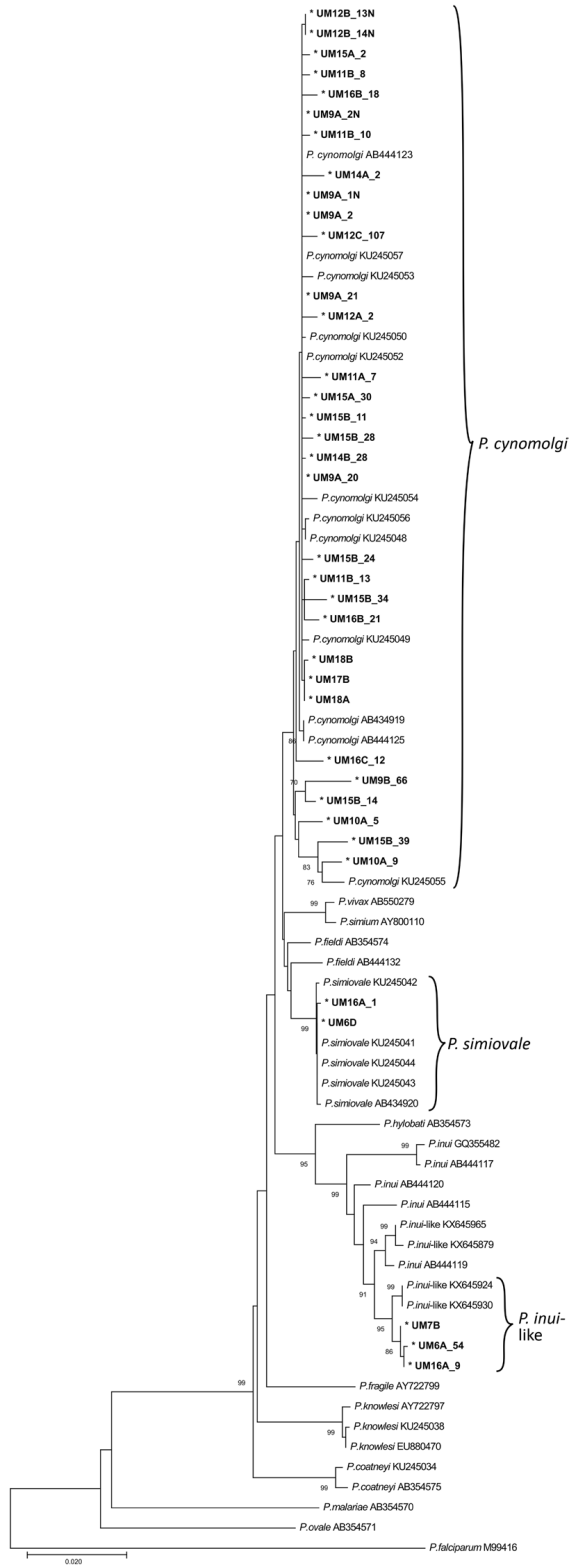
Neighbor-joining phylogenetic tree of *Plasmodium* species based on partial sequence of COX1 genes for identification of *Plasmodium* malaria species from indigenous community blood samples, Malaysia. Nucleotide sequences generated from this study are marked with asterisks and are in bold. GenBank accession numbers are provided for all sequences. Numbers at nodes indicate percentage support of 1,000 bootstrap replicates; only bootstrap values above 70% are displayed. Scale bar indicates branch length.

We then subjected the PCR-positive samples (UM6–7, UM9–12, UM14–18) to amplification and sequencing of partial COX1 genes. Neighbor-joining (Figure 2) phylogenetic inference of these sequences, together with available referral sequences from GenBank, indicated that 32 haplotypes from samples UM9–12 and UM14–18 were genetically indistinguishable from *P. cynomolgi*. Our phylogenetic analyses also demonstrated that sample UM7 had a single infection with *P. inui*–like parasites, whereas UM6 had a double infection with *P. simiovale* and *P. inui*–like parasites and UM16 had a triple infection with *P. cynomolgi, P. simiovale*, and *P. inui*–like parasites. 

We generated phylogenetic trees of similar topology by the maximum-likelihood method for the SSU rRNA genes (Appendix Figure 1) and by the Bayesian maximum clade credibility method for the COX1 genes (Appendix Figure 2). There were discrepancies between the nested PCR assay results and the sequencing results between our 2 laboratories; mixed species of *Plasmodium* were identified only at UNIMAS. A possible explanation is that the DNA samples analyzed at UNIMAS were newly extracted and were different from the ones used in the experiments at UM. There might also be a compromise of the sensitivity in detecting the species with lower parasitemia in mixed infections as a result of competition for nest 1 primers by the species with higher parasite loads. Furthermore, for sequencing of the SSU rRNA genes at UM, primers that were specific for the species identified by nested PCR assays were used, whereas for the COX1 genes, both *P. cynomolgi*–specific primers and primers that could amplify other species of *Plasmodium* were used. Therefore, additional species of *Plasmodium* were identified at UNIMAS in these samples, such as *P. simiovale* and *P. inui*–like, for which no species-specific PCR primers exist.

## Conclusions

The 40 *P. knowlesi* infections we detected originated from 6 states in Malaysia, thereby confirming the widespread distribution of human *P. knowlesi* malaria cases in Malaysia (*1*). We detected *P. cynomolgi* infections among indigenous communities in 4 states in Malaysia. Taken together with previous reports of naturally acquired *P. cynomolgi* infections in humans in the states of Terengganu, Sabah, and Sarawak (*3*,*8*,*9*), our findings indicate that human infections caused by *P. cynomolgi* are also widely distributed in Malaysia.

Our study highlights the occurrence of naturally acquired human infections with *P. inui, P. inui*–like, *P. coatneyi*, and *P. simiovale*. Natural human *P. inui* infections have not been described (*10*), although the parasite is experimentally transmissible to humans (*2*). For *P. coatneyi*, attempts to infect humans with blood from an infected rhesus monkey and through infected mosquitoes were unsuccessful (*2*). *P. simiovale* is a lesser-studied simian malaria parasite that was previously described only in toque macaques (*Macaca sinica*) of Sri Lanka (*2*) until it was recently identified, together with *P. inui*–like parasites, in long-tailed macaques from Sarawak in Malaysian Borneo (*11*). All these simian malaria parasites would have been diagnosed by microscopy as human malaria parasites because they share morphological similarities with human malaria parasites. The early blood stages of *P. knowlesi* resemble those of *P. falciparum*, and the other forms are similar to *P. malariae* (*2*,*6*). *P. cynomolgi* is morphologically similar to *P. vivax* (*2*), and both *P. inui* and *P. inui*–like parasites are morphologically identical to *P. malariae* (*2*,*11*), whereas *P. coatneyi* bears morphologic similarities to *P. falciparum* and *P. simiovale* bears morphologic similarities to *P. ovale* (*2*,*12*). Besides misdiagnosis of simian malaria parasites as human malaria parasites, there are other limitations of microscopy for diagnosis of malaria; thus, utilization of molecular tools is paramount in generating accurate epidemiology data (*6*). It is envisaged that screening with molecular tools of other communities living at the forest fringes will demonstrate the widespread distribution of zoonotic malaria and uncover more newly emergent malaria parasites.

Appendix Additional information about human infections with simian malaria parasites in Malaysia.
