# Supplementary material for: Natural Human Infections with Plasmodium cynomolgi, P. inui, and 4 other Simian Malaria Parasites, Malaysia
Source: Emerg Infect Dis. 2021 Aug;27(8):2187–91. doi: 10.3201/eid2708.204502 (PMC8314832; doi:10.3201/eid2708.204502)
Supplement: Appendix — Additional information about human infections with simian malaria parasites in Malaysia. [file 20-4502-Techapp-s1.pdf]

Article DOI: https://doi.org/10.3201/eid2708.204502

Natural Human Infections with *Plasmodium cynomolgi*, *P. inui*, and 4 other Simian Malaria Parasites, Malaysia

Appendix

Materials and Methods

Ethical Considerations

The study was approved by the Medical Research Ethics Committee of University of Malaya Medical Centre (MEC Ref. No. 920.83). Approval was also obtained from the Department of Orang Asli (Indigenous) Development and the respective heads of the villages before blood sample collection from the indigenous communities. We obtained informed consents from those who agreed to participate, or from parents on behalf of their children.

Source of Archived Blood Samples

We examined 645 archived blood samples that we had collected during 2011–2014 among indigenous populations of various subtribes from 14 villages in 7 states of Malaysia: Pahang, Perak, Selangor, Negeri Sembilan, Melaka, Kelantan, and Sarawak (Appendix Table 1). These indigenous community samples were obtained during previous studies focusing on intestinal parasites. Therefore, information such as body temperature, malaria history, and malaria parasite density were not available.

The indigenous communities we studied here are a diverse group. There are ≥95 subgroups distributed in selected states throughout Malaysia, each with its own distinct language and culture. The indigenous population of peninsular Malaysia is separated into 3 main tribal groups, Negrito, Senoi, and Proto Malay (Aboriginal Malay), and consists of 18 subtribes. The largest indigenous groups in Malaysian Borneo are Ibans in Sarawak and the Kadazan Dusuns in Sabah. The indigenous communities that we studied all live in the forest fringe and are engaged with forest and agricultural activities in which there is a greater chance of being exposed to the macaque reservoirs and mosquito vectors (*1*,*2*).

Molecular Detection of *Plasmodium* Species at Universiti Malaya (UM)

We extracted genomic DNA from either blood (≈3 mL) or blood spots on filter paper using the QIAamp DNA Blood Mini Kit (QIAGEN, https://www.qiagen.com), according to the manufacturer's instructions, and stored the samples at −20°C until further analysis. We first screened the DNA samples at UM for the presence of *Plasmodium* with the aid of genus-specific primers (rPLU1, rPLU5, rPLU3 and rPLU4), as described previously (*3*). We then examined *Plasmodium*-positive samples by nested PCR assays using species-specific primers for *Plasmodium falciparum*, *P. vivax*, *P. malariae, P. ovale curtisi*, *P. ovale wallikeri* (*3*), *P. knowlesi* (*4*), *P. coatneyi*, *P. cynomolgi*, *P. inui*, and *P. fieldi* (*5*).

Amplification and Sequencing of SSU rRNA Genes of *Plasmodium* Species at UM

We amplified and sequenced longer fragments of SSU rRNA genes of simian *Plasmodium* species (914–950 bp) by nested PCR assays with other pairs of species-specific primers (*6*). We performed PCR amplifications in a 50 µL reaction volume consisting of 5 µL DNA template from previously amplified PCR product, 1X PCR buffer (Promega, https://www.promega.com), 0.2 mM dNTPs, 3 mM $MgCl_2$, 1.5 U Taq DNA polymerase, and 0.5 µM forward and reverse primers. The PCR was carried out in a MyCycler Thermal Cycler (Bio-Rad, https://www.bio-rad.com) under the following conditions: 94°C for 5 min; 35 cycles of 94°C for 1 min, 50–60°C for 90 sec, 72°C for 1 min; 72°C for 10 min. We examined all PCR products (1015–1050 bp) using 1.5% agarose gels before we sent amplicons to a commercial facility for bidirectional sequencing (BigDye Terminator v.3.1 chemistry; Applied Biosystems, https://www.thermofisher.com).

Molecular Detection of Simian *Plasmodium* Species at Universiti Malaysia Sarawak (UNIMAS)

We subsequently extracted DNA from 15 blood samples we had identified as having *P. cynomolgi*, *P. coatneyi* and *P. inui*, and 5 samples that were malaria-negative at UM. We then sent these samples blind to Universiti Malaysia Sarawak (UNIMAS), where they were first examined by nested PCR assays for *Plasmodium*, and the *Plasmodium*-positive ones were examined with species-specific primers as described previously (*3,5,7*).

PCR Amplification and Sequencing COX1 Genes at UNIMAS

Sequencing of the partial COX1 genes of *Plasmodium* involved a single-step PCR or a hemi-nested PCR. We amplified 3 samples (UM10, UM11, UM14) with single-step PCR and 3

(UM6, UM7, UM18) with hemi-nested PCR; we used both methods for 4 (UM9, UM12, UM15, UM16).

In the hemi-nested PCR, we amplified the complete COX1 gene using *Plasmodium*-specific primers: CYFinF1 (5′-CCTGACATGGATGGATAATACTCG-3′) and CYFinR2 (5′-CCATCCATTTAAAGCGTCTGG-3′). We performed Nest 1 PCR amplification in a 50 μL reaction mixture containing 1× Colorless GoTaq PCR buffer, 2.5 mmol of $MgCl_2$, 0.2 mmol dNTP mix (Promega, https://www.promega.com), 0.025 U GoTaq DNA polymerase, 0.25 μmol of each primer (CYFinF1 and CYFinR2), and 5 μL of purified genomic DNA under the following conditions: 94°C for 4 min; 30 cycles of 94°C for 1 min, 53°C for 1 min, and 72°C for 90 sec; 5 min at 72°C. We then used the Nest 1 amplicons as a template for the hemi-nested PCR assay with *P. cynomolgi*-specific primers: cox1_F1 (5′-CCAAGCCTCACTTATTGTTAAT-3′) (*8*) and CYFinR2 and *Plasmodium*-specific primers: CYFinF3 (5′-CCAAAGTATAACCGCTGTCGC-3′) and CYFinR2. We performed the hemi-nested PCR amplification for each sample in a 30 μL reaction mixture containing 1× HF colorless PCR buffer, 0.2 mmol dNTP mix, 0.02 U Phusion Polymerase (Promega), 0.5 μmol of each primer (cox1_F1 and CYFinR2 or CYFinF3 and CYFinR2), and 3 μL of Nest 1 product under the following conditions: 98°C for 30 sec; 35 cycles at 98°C for 7 sec, 60°C (for cox1_F1 and CYFinR2) and 62°C (for CYFinF3 and CYFinR2) for 20 sec, and 72°C for 22 sec; and 72°C for 10 min.

We performed single-step PCR amplification of *P. cynomolgi* COX1 fragment using *P. cynomolgi*-specific primers: cox1_F1 (5′- CCAAGCCTCACTTATTGTTAAT-3′) and cox1_R1 (5′- ACCAAATAAAGTCATTGTTGATCC-3′) (*8*). We performed amplifications in a 30 μL reaction mixture containing similar concentrations of PCR master-mix components with cox1_F1 and CYFinR2 or with CYFinF3 and CYFinR2 primers and 3 μL of purified genomic DNA as the template, using the following parameters: 98°C for 30 sec; 35 cycles at 98°C for 7 sec, 58°C for 20 sec, and 72°C for 28 sec; and 72°C for 10 min.

We performed *Plasmodium sp.* DNA cloning and transformation of the recombinant plasmids using the Zero Blunt TOPO PCR Cloning Kit, with One Shot TOP10 Chemically Competent *E. coli* cells (Invitrogen, https://www.thermofisher.com). We extracted plasmid DNA

using the PureLink Quick Plasmid DNA Miniprep Kit (Invitrogen) and sent plasmids to a commercial facility for bidirectional DNA sequencing.

Phylogenetic Analysis

We trimmed and aligned the SSU rRNA sequences of *Plasmodium* species using the Geneious version 9.1.6 software (*9*). We constructed phylogenetic trees using the neighbor-joining method as described in MEGA v10.0.5 software (*10*) with bootstrap percentage based on 1,000 replications. We deposited the sequences in GenBank under accession nos. MK351344–MK351383, MK351405–MK351407, MK351409–MK351417, and MK351420–MK351422 (Appendix Table 2).

We used ClustalX v2 to align the partial COX1 sequences. We inferred phylogenetic relationships using the neighbor-joining method (*11*) implemented in MEGA v10.0.5. We reconstructed the neighbor-joining tree with 1,000 bootstrap percentage based on 1,000 replications. We used Tree Annotator to annotate the tree generated by BEAST (https://www.mybiosoftware.com) and visualized the maximum clade credibility tree using FigTree v1.3.1 (https://figtree-1-3-1.software.informer.com). We deposited the *Plasmodium* COX1 sequences generated in GenBank under accession nos. MT992662-MT992702 (Appendix Table 3).

Molecular Detection of Macaque DNA in the Human Samples at UNIMAS

We screened all 20 samples sent from UM to UNIMAS for the presence of macaque DNA. We amplified the cytochrome c oxidase subunit 1 (COX1) gene by PCR using *Macaca* genus-specific primers MacF (5′-CAACGTYATYGTAACGGC-3′) and MacR (5′-AGGTAGTATTGAGGTTGC-3′). We performed Nest 1 PCR amplification for each sample using the Applied Biosystems ProFlex PCR System thermocycler (Thermo Fisher Scientific, https://www.thermofisher.com) in a 20 μL reaction mixture containing 1× colorless GoTaq PCR buffer (Promega), 2 mmol of $MgCl_2$, 0.2 mmol dNTP mix, 0.25 μmol of each primer (MacF and MacR), 0.025 U GoTaq DNA polymerase, and 2 μL of purified genomic DNA under the following conditions: 94°C for 4 min; 35 cycles of 94°C for 30 sec, 59°C for 1 min, and 72°C for 30 sec; and 72°C for 5 min. We used *M. fascicularis*-specific primers MfF (5′-AGGGTTCGGGAACTGACTG-3′) and MfR (5′-TGATCAGACAAATAAGGGGTC-3′) and *M. nemestrina*-specific primers MnF (5′-CATACCTATTATGATTGGGGGT-3′) and MnR (5′-

GGTGGAGGGAGAAGATGATTAGG-3′) for subsequent PCR amplification in a 20 μL reaction mixture containing 1× Green GoTaq PCR buffer (Promega), 2 mmol of $MgCl_2$, 0.2 mmol dNTP mix, 0.25 μmol of each primer (MfF and MfR or MnF and MnR), and 0.025 U GoTaq DNA polymerase with 2 μL of Nest 1 product under the following conditions: 94°C for 4 min; 35 cycles of 94°C for 30 sec, 57°C for 1 min, and 72°C for 30 sec; and 72°C for 5 min.

References

1. Jiram AI, Hisam S, Reuben H, Husin SZ, Roslan A, Wan Ismail WR. Submicroscopic evidence of the simian malaria parasite, *Plasmodium knowlesi*, in an Orang Asli community. Southeast Asian J Trop Med Public Health. 2015;47:4.

2. Kaur G. Prevalence of clinical malaria among an Orang Asli community in Malaysia. Southeast Asian J Trop Med Public Health. 2009;40:665–73. PubMed

3. Singh B, Bobogare A, Cox-Singh J, Snounou G, Abdullah MS, Rahman HA. A genus- and species-specific nested polymerase chain reaction malaria detection assay for epidemiologic studies. Am J Trop Med Hyg. 1999;60:687–92. PubMed https://doi.org/10.4269/ajtmh.1999.60.687

4. Singh B, Sung LK, Matusop A, Radhakrishnan A, Shamsul SS, Cox-Singh J, et al. A large focus of naturally acquired *Plasmodium knowlesi* infections in human beings. Lancet. 2004;363:1017–24. PubMed https://doi.org/10.1016/S0140-6736(04)15836-4

5. Lee KS, Divis PC, Zakaria SK, Matusop A, Julin RA, Conway DJ, et al. *Plasmodium knowles*i: reservoir hosts and tracking the emergence in humans and macaques. PLoS Pathog. 2011;7:e1002015. PubMed https://doi.org/10.1371/journal.ppat.1002015

6. Chua TH, Manin BO, Daim S, Vythilingam I, Drakeley C. Phylogenetic analysis of simian *Plasmodium* spp. infecting *Anopheles balabacensis Baisas* in Sabah, Malaysia. PLoS Negl Trop Dis. 2017;11:e0005991. PubMed https://doi.org/10.1371/journal.pntd.0005991

7. Snounou G, Viriyakosol S, Zhu XP, Jarra W, Pinheiro L, do Rosario VE, et al. High sensitivity of detection of human malaria parasites by the use of nested polymerase chain reaction. Mol Biochem Parasitol. 1993;61:315–20. PubMed https://doi.org/10.1016/0166-6851(93)90077-B

8. Raja TN, Hu TH, Kadir KA, Mohamad DSA, Divis PCS, Wong LL, et al. Naturally acquired human infections with *Plasmodium cynomolgi* and *Plasmodium knowlesi* infections, Malaysian Borneo. Emerg Infect Dis. 2020;26:1801–9. PubMed https://doi.org/10.3201/eid2608.200343

9. Kearse M, Moir R, Wilson A, Stones-Havas S, Cheung M, Sturrock S, et al. Geneious Basic: an integrated and extendable desktop software platform for the organization and analysis of sequence data. Bioinformatics. 2012;28:1647–9. PubMed https://doi.org/10.1093/bioinformatics/bts199

10. Kumar S, Stecher G, Li M, Knyaz C, Tamura K. MEGA X: Molecular evolutionary genetics analysis across computing platforms. Mol Biol Evol. 2018;35:1547–9. PubMed https://doi.org/10.1093/molbev/msy096

11. Saitou N, Nei M. The neighbor-joining method: a new method for reconstructing phylogenetic trees. Mol Biol Evol. 1987;4:406–25. PubMed

12. Choy SH, Al-Mekhlafi HM, Mahdy MA, Nasr NN, Sulaiman M, Lim YA, et al. Prevalence and associated risk factors of *Giardia* infection among indigenous communities in rural Malaysia. Sci Rep. 2014;4:6909. PubMed https://doi.org/10.1038/srep06909

13. Lee SC, Tang MS, Lim YA, Choy SH, Kurtz ZD, Cox LM, et al. Helminth colonization is associated with increased diversity of the gut microbiota. PLoS Negl Trop Dis. 2014;8:e2880. PubMed https://doi.org/10.1371/journal.pntd.0002880

14. Rajoo Y, Ambu S, Lim YA, Rajoo K, Tey SC, Lu CW, et al. Neglected intestinal parasites, malnutrition and associated key factors: a population based cross-sectional study among indigenous communities in Sarawak, Malaysia. PLoS One. 2017;12:e0170174. PubMed https://doi.org/10.1371/journal.pone.0170174

Appendix Table 1. Distribution of indigenous community blood samples used in the study of *Plasmodium* infections in Malaysia, according to state, district, village, and subtribe (N = 645).

State	District	Village	Subtribe	n (%)	Reference
Peninsular Malaysia					
Pahang	Pekan	Chini	Proto-Malay (Jakun)	9 (1.4)	Unpublished data
	Temerloh	Paya Sendayan	Senoi (Jahut)	97 (15.0)	(*12*)
	Lanchang	Kuala Gandah	Senoi (Che Wong)	3 (0.5)	Unpublished data
Perak	Slim River	Sungail Bil	Senoi (Semai)	40 (6.2)	Unpublished data
	Tapah	Batu 7 1/2	Senoi (Semai)	7 (1.1)	Unpublished data
		Batu 8	Senoi (Semai)	14 (2.2)	Unpublished data
Selangor	Semenyih	Donglai Baru	Proto-Malay (Temuan)	49 (7.6)	(*12*)
Negeri Sembilan	Jelebu	Dusun Kubur	Proto-Malay (Temuan)	100 (15.5)	(*13*)
		Ulu Kelaka	Proto-Malay (Temuan)	63 (9.8)	(*13*)
Melaka	Alor Gajah	Bukit Sebang	Proto-Malay (Temuan)	9 (1.4)	(*12*)
		Bukit Payung	Proto-Malay (Temuan)	23 (3.6)	(*12*)
Kelantan	Gua Musang	Kuala Lah	Negrito (Mendriq)	15 (2.3)	Unpublished data
		Aring 5	Negrito (Bateq)	17 (2.6)	Unpublished data
Malaysia Borneo					
Sarawak	Sarikei	Pakan	Iban	199 (30.9)	(*14*)

Appendix Table 2. GenBank accession numbers of partial sequence SSU rRNA gene generated from simian *Plasmodium* species found in study at Universiti Malaya

GenBank accession no.	*Plasmodium* spp.	Location	Sample ID
MK351344	*P. knowlesi*	Pakan, Sarikei, Sarawak	PK1
MK351345	*P. knowlesi*	Pakan, Sarikei, Sarawak	PK2
MK351346	*P. knowlesi*	Pakan, Sarikei, Sarawak	PK3
MK351347	*P. knowlesi*	Kg Kuala Gandah, Lanchang, Pahang	PK4
MK351348	*P. knowlesi*	Kg Chini, Pekan, Pahang	PK5
MK351349	*P. knowlesi*	Kg Ulu Kelaka, Jelebu, Negeri Sembilan	PK6
MK351350	*P. knowlesi*	Kg Ulu Kelaka, Jelebu, Negeri Sembilan	PK7
MK351351	*P. knowlesi*	Kg Sungai Bil, Slim River, Perak	PK8
MK351352	*P. knowlesi*	Kg Sungai Bil, Slim River, Perak	PK9
MK351353	*P. knowlesi*	Kg Sungai Bil, Slim River, Perak	PK10
MK351354	*P. knowlesi*	Kg Sungai Bil, Slim River, Perak	PK11
MK351355	*P. knowlesi*	Kg Sungai Bil, Slim River, Perak	PK12
MK351356	*P. knowlesi*	Kg Sungai Bil, Slim River, Perak	PK13
MK351357	*P. knowlesi*	Kg Sungai Bil, Slim River, Perak	PK14
MK351358	*P. knowlesi*	Kg Sungai Bil, Slim River, Perak	PK15
MK351359	*P. knowlesi*	Kg Sungai Bil, Slim River, Perak	PK16
MK351360	*P. knowlesi*	Kg Sungai Bil, Slim River, Perak	PK17
MK351361	*P. knowlesi*	Kg Sungai Bil, Slim River, Perak	PK18
MK351362	*P. knowlesi*	Kg Sungai Bil, Slim River, Perak	PK19
MK351363	*P. knowlesi*	Kg Sungai Bil, Slim River, Perak	PK20
MK351364	*P. knowlesi*	Kg Sungai Bil, Slim River, Perak	PK21
MK351365	*P. knowlesi*	Kg Sungai Bil, Slim River, Perak	PK22
MK351366	*P. knowlesi*	Kg Batu 7 1/2, Tapah, Perak	PK23
MK351367	*P. knowlesi*	Kg Batu 7 1/2, Tapah, Perak	PK24
MK351368	*P. knowlesi*	Kg Batu 7 1/2, Tapah, Perak	PK25
MK351369	*P. knowlesi*	Kg Batu 7 1/2, Tapah, Perak	PK26
MK351370	*P. knowlesi*	Kg Batu 8, Tapah, Perak	PK27
MK351371	*P. knowlesi*	Kg Batu 8, Tapah, Perak	PK28
MK351372	*P. knowlesi*	Kg Batu 8, Tapah, Perak	PK29
MK351373	*P. knowlesi*	Kg Batu 8, Tapah, Perak	PK30
MK351374	*P. knowlesi*	Kg Batu 8, Tapah, Perak	PK31
MK351375	*P. knowlesi*	Kg Batu 8, Tapah, Perak	PK32
MK351376	*P. knowlesi*	Kg Batu 8, Tapah, Perak	PK33
MK351377	*P. knowlesi*	Kg Bukit Sebang, Alor Gajah, Melaka	PK34
MK351378	*P. knowlesi*	Kg Kuala Lah, Gua Musang, Kelantan	PK35
MK351379	*P. knowlesi*	Kg Kuala Lah, Gua Musang, Kelantan	PK36
MK351380	*P. knowlesi*	Kg Kuala Lah, Gua Musang, Kelantan	PK37
MK351381	*P. knowlesi*	Kg Aring 5, Gua Musang, Kelantan	PK38
MK351382	*P. knowlesi*	Kg Aring 5, Gua Musang, Kelantan	PK39
MK351383	*P. knowlesi*	Kg Aring 5, Gua Musang, Kelantan	PK40
MK351405	*P. coatneyi*	Kg Sungai Bil, Slim River, Perak	UM1
MK351406	*P. coatneyi*	Kg Batu 7 1/2, Tapah, Perak	UM2
MK351407	*P. coatneyi*	Kg Batu 8, Tapah, Perak	UM3
MK351409	*P. cynomolgi*	Kg Sungai Bil, Slim River, Perak	UM9
MK351410	*P. cynomolgi*	Kg Sungai Bil, Slim River, Perak	UM11
MK351411	*P. cynomolgi*	Kg Sungai Bil, Slim River, Perak	UM15
MK351412	*P. cynomolgi*	Kg Batu 8, Tapah, Perak	UM17
MK351413	*P. cynomolgi*	Kg Batu 8, Tapah, Perak	UM12
MK351414	*P. cynomolgi*	Kg Ulu Kelaka, Jelebu, Negeri Sembilan	UM14
MK351415	*Plasmodium* spp.	Kg Ulu Kelaka, Jelebu, Negeri Sembilan	UM16
MK351416	*P. cynomolgi*	Kg Bukit Sebang, Alor Gajah, Melaka	UM18
MK351420	*P. inui*	Kg Bukit Sebang, Alor Gajah, Melaka	UM5
MK351421	*P. inui*	Pakan, Sarikei, Sarawak	UM6
MK351422	*P. inui*	Pakan, Sarikei, Sarawak	UM7

Appendix Table 3. GenBank accession numbers of partial sequence COX1 gene generated from simian *Plasmodium* species found in study at Universiti Malaysia Sarawak

GenBank accession no.	*Plasmodium* spp.	Location	Clone identity*
MT992662	*P. cf. inui*	Pakan, Sarikei, Sarawak	UM6A_54
MT992663	*P. simiovale*	Pakan, Sarikei, Sarawak	UM6D
MT992664	*P. cf. inui*	Pakan, Sarikei, Sarawak	UM7B
MT992665	*P. cynomolgi*	Kg Sungai Bil, Slim River, Perak	UM9A_1N
MT992666	*P. cynomolgi*	Kg Sungai Bil, Slim River, Perak	UM9A_2N
MT992667	*P. cynomolgi*	Kg Sungai Bil, Slim River, Perak	UM9A_2
MT992668	*P. cynomolgi*	Kg Sungai Bil, Slim River, Perak	UM9A_20
MT992669	*P. cynomolgi*	Kg Sungai Bil, Slim River, Perak	UM9A_21
MT992670	*P. cynomolgi*	Kg Sungai Bil, Slim River, Perak	UM9B_66
MT992671	*Plasmodium sp.*	Kg Sungai Bil, Slim River, Perak	UM9B_75
MT992672	*P. cynomolgi*	Kg Aring 5, Gua Musang, Kelantan	UM10A_5
MT992673	*P. cynomolgi*	Kg Aring 5, Gua Musang, Kelantan	UM10A_9
MT992674	*P. cynomolgi*	Kg Sungai Bil, Slim River, Perak	UM11A_7
MT992675	*P. cynomolgi*	Kg Sungai Bil, Slim River, Perak	UM11B_8
MT992676	*P. cynomolgi*	Kg Sungai Bil, Slim River, Perak	UM11B_10
MT992677	*P. cynomolgi*	Kg Sungai Bil, Slim River, Perak	UM11B_13
MT992678	*P. cynomolgi*	Kg Batu 8, Tapah, Perak	UM12A_2
MT992679	*P. cynomolgi*	Kg Batu 8, Tapah, Perak	UM12B_13N
MT992680	*P. cynomolgi*	Kg Batu 8, Tapah, Perak	UM12B_14N
MT992681	*P. cynomolgi*	Kg Batu 8, Tapah, Perak	UM12C_107
MT992682	*P. cynomolgi*	Kg Ulu Kelaka, Jelebu, Negeri Sembilan	UM14A_2
MT992683	*P. cynomolgi*	Kg Ulu Kelaka, Jelebu, Negeri Sembilan	UM14B_28
MT992684	*P. cynomolgi*	Kg Sungai Bil, Slim River, Perak	UM15A_2
MT992685	*P. cynomolgi*	Kg Sungai Bil, Slim River, Perak	UM15A_30
MT992686	*Plasmodium sp.*	Kg Sungai Bil, Slim River, Perak	UM15B_9
MT992687	*P. cynomolgi*	Kg Sungai Bil, Slim River, Perak	UM15B_11
MT992688	*Plasmodium sp.*	Kg Sungai Bil, Slim River, Perak	UM15B_12
MT992689	*Plasmodium sp.*	Kg Sungai Bil, Slim River, Perak	UM15B_13
MT992690	*P. cynomolgi*	Kg Sungai Bil, Slim River, Perak	UM15B_14
MT992691	*P. cynomolgi*	Kg Sungai Bil, Slim River, Perak	UM15B_24
MT992692	*P. cynomolgi*	Kg Sungai Bil, Slim River, Perak	UM15B_28
MT992693	*P. cynomolgi*	Kg Sungai Bil, Slim River, Perak	UM15B_34
MT992694	*P. cynomolgi*	Kg Sungai Bil, Slim River, Perak	UM15B_39
MT992695	*P. simiovale*	Kg Ulu Kelaka, Jelebu, Negeri Sembilan	UM16A_1
MT992696	*P. cf. inui*	Kg Ulu Kelaka, Jelebu, Negeri Sembilan	UM16A_9
MT992697	*P. cynomolgi*	Kg Ulu Kelaka, Jelebu, Negeri Sembilan	UM16B_18
MT992698	*P. cynomolgi*	Kg Ulu Kelaka, Jelebu, Negeri Sembilan	UM16B_21
MT992699	*P. cynomolgi*	Kg Ulu Kelaka, Jelebu, Negeri Sembilan	UM16C_12
MT992700	*P. cynomolgi*	Kg Batu 8, Tapah, Perak	UM17B
MT992701	*P. cynomolgi*	Kg Bukit Sebang, Alor Gajah, Melaka	UM18A
MT992702	*P. cynomolgi*	Kg Bukit Sebang, Alor Gajah, Melaka	UM18B

*Clone identity is the identity of the clones derived from samples UM 6–7, UM 9–12, and UM 14–18.

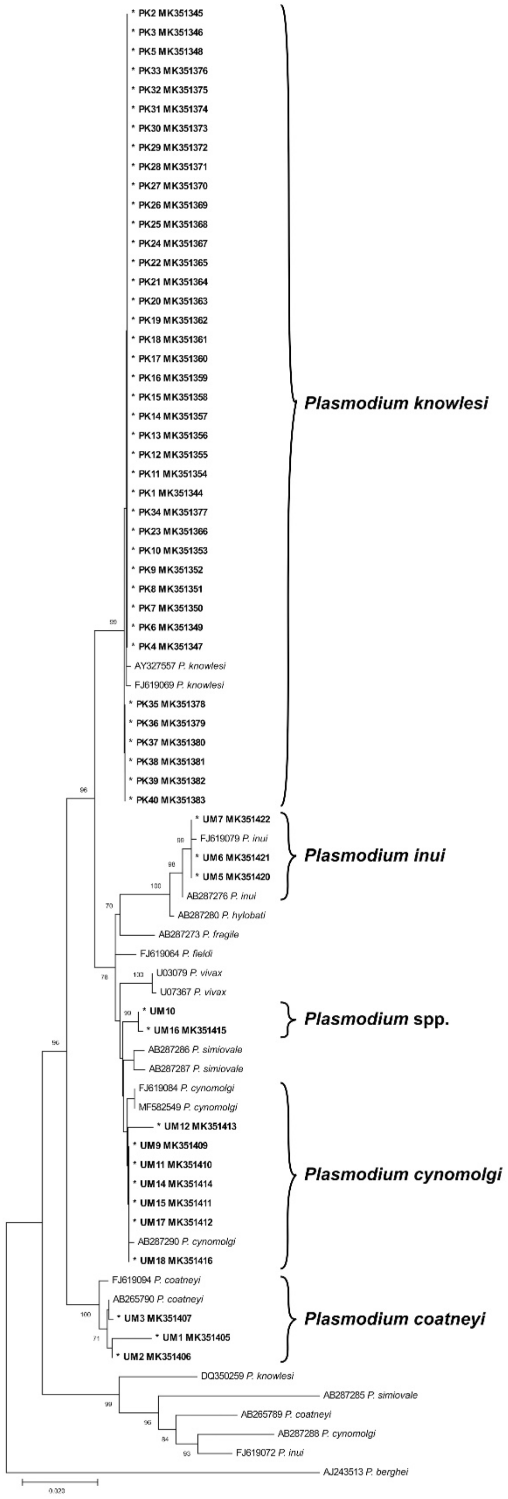

Appendix Figure 1. Maximum-likelihood phylogenetic tree of *Plasmodium* species, based on partial sequence of SSU rRNA genes. Numbers at nodes indicate percentage support of 1,000 bootstrap replicates; only bootstrap values above 70% are displayed. Nucleotide sequences generated from our study are marked with asterisks and are in bold. Scale bar indicates branch length.

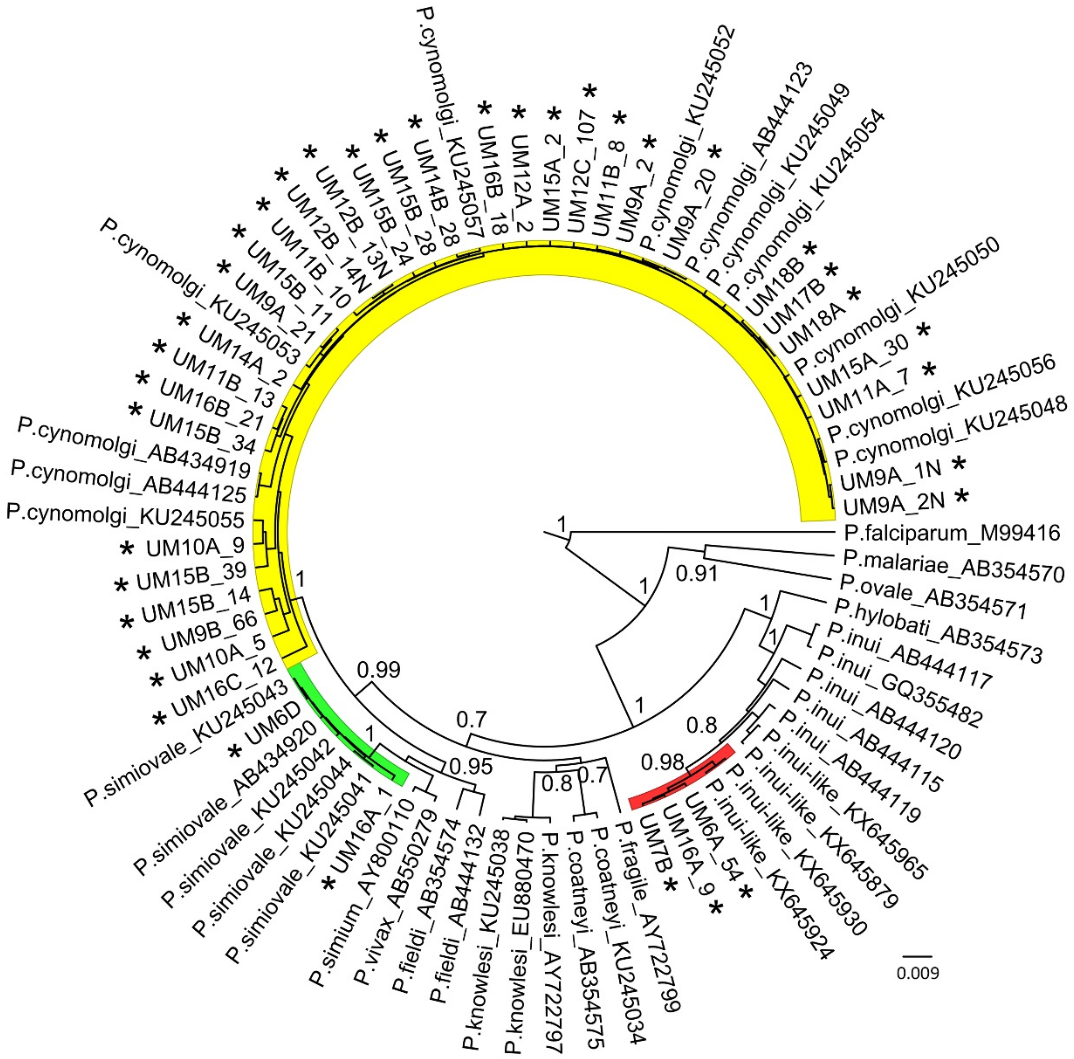

Appendix Figure 2. Maximum clade credibility phylogeny of *Plasmodium* species based on partial sequence of COX1 genes inferred using the Bayesian method. Numbers on branches are values of posterior probabilities. Sequences of *P. cynomolgi* are highlighted in yellow, *P. simiovale* in green, and *P. inui*-like in red. Nucleotide sequences generated from the present study are marked with asterisks.
